# Chaperoning Proteins for Destruction: Diverse Roles of Hsp70 Chaperones and their Co-Chaperones in Targeting Misfolded Proteins to the Proteasome

**DOI:** 10.3390/biom4030704

**Published:** 2014-07-17

**Authors:** Ayala Shiber, Tommer Ravid

**Affiliations:** Department of Biological Chemistry, The Alexander Silberman Institute of Life Sciences, The Hebrew University of Jerusalem, Edmond J. Safra Campus, Givat-Ram, Jerusalem 91904, Israel; E-Mail: shibera@gmail.com

**Keywords:** the Ub-proteasome system, molecular chaperones, protein misfolding, protein degradation, protein aggregation, yeast, Hsp40, Hsp70

## Abstract

Molecular chaperones were originally discovered as heat shock-induced proteins that facilitate proper folding of proteins with non-native conformations. While the function of chaperones in protein folding has been well documented over the last four decades, more recent studies have shown that chaperones are also necessary for the clearance of terminally misfolded proteins by the Ub-proteasome system. In this capacity, chaperones protect misfolded degradation substrates from spontaneous aggregation, facilitate their recognition by the Ub ligation machinery and finally shuttle the ubiquitylated substrates to the proteasome. The physiological importance of these functions is manifested by inefficient proteasomal degradation and the accumulation of protein aggregates during ageing or in certain neurodegenerative diseases, when chaperone levels decline. In this review, we focus on the diverse roles of stress-induced chaperones in targeting misfolded proteins to the proteasome and the consequences of their compromised activity. We further discuss the implications of these findings to the identification of new therapeutic targets for the treatment of amyloid diseases.

## 1. Introduction

The cell’s capacity to maintain protein homeostasis is constantly challenged, as proteins can often fail to attain or preserve their native conformation due to destabilizing mutations, translation errors, stress conditions or developmental changes [1]. Failure to sustain cellular protein homeostasis leads to the accumulation of misfolded proteins and to the formation of insoluble aggregates that can be toxic and ultimately even induce cell death. Protein conformation diseases such as Alzheimer’s, Parkinson’s and Huntington’s underscore the importance of high fidelity protein quality-control (PQC) mechanisms for cell survival.

**Figure 1:**
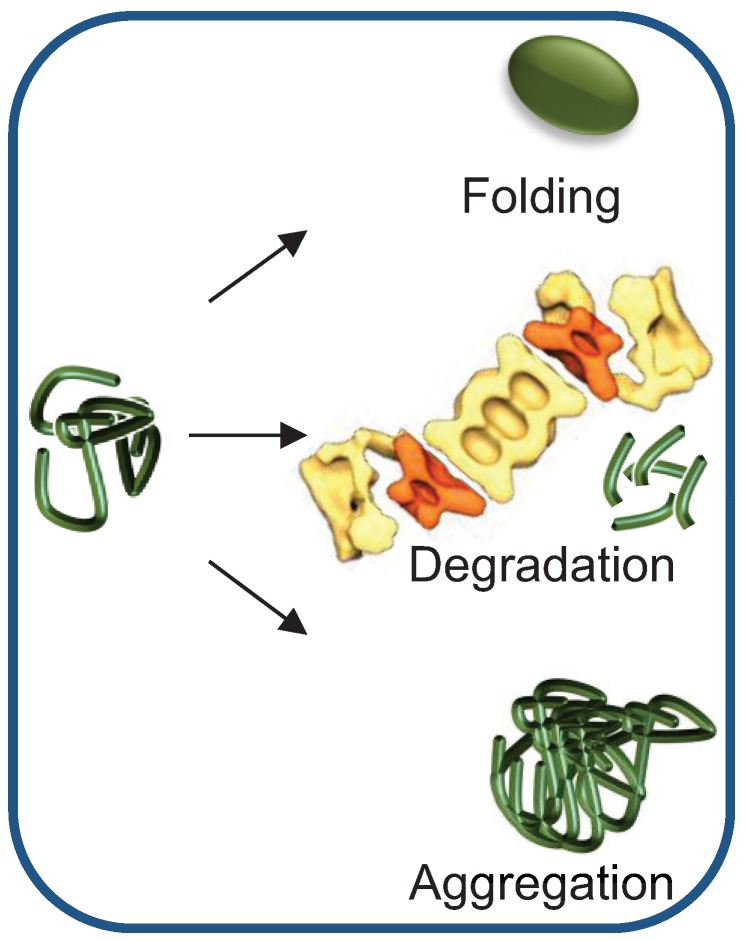
Misfolded protein quality control: making the correct triage decision. Preferentially, the misfolded protein is refolded, keeping the active protein pool constant. However, if the protein cannot refold to its native conformation, it can be removed by Ub-dependent proteasomal degradation. If the chaperone and/or the degradation machineries become rate limiting, misfolded proteins are likely to aggregate. Both degradation and aggregation reduce the active pool of the protein. The proteasome cartoon was adapted from Nickell *et al.* [3].

In order to maintain balanced protein homeostasis, several protective mechanisms that deal with aberrant proteins have evolved. These highly evolutionary conserved mechanisms are employed depending on the extent of protein misfolding and on the capacity of the PQC network (Figure 1). Clearly, the preferred means of dealing with a misfolded protein is to refold it. However, if refolding fails, the protein is disposed of through proteolysis by the ubiquitin (Ub) proteasome system (UPS), the main proteolytic system in eukaryotic cells. If both refolding and proteasomal degradation activities are inhibited or otherwise overwhelmed, the misfolded protein can be sequestered into designated inclusion bodies. These inclusions may be then degraded by selective autophagy, a lysosome-mediated degradation pathway [2]. A hallmark of all of these pathways is the requirement for heat shock proteins (HSPs), a family of molecular chaperones, induced as part of the cell heat shock response.

The heat shock response was discovered over 50 years ago by Ritossa and colleagues, who observed a distinct chromosomal “puffing” pattern in *Drosophila* flies in response to heat shock [4]. This phenomenon, indicative of a massive transcriptional activation [5], was found to be accompanied by *de-novo* synthesis of a group of proteins, termed accordingly “Heat Shock Proteins” [6,7]. Since then, it has been found that HSPs are upregulated in response to a variety of stimuli and some are relatively abundant even under normal conditions [8,9].

The HSPs are a set of functionally related proteins, which are classified primarily according to their molecular weight: HSP40, HSP60, HSP70, HSP90, HSP100 and small HSPs [10]. The basic function of the HSPs is to selectively recognize non-native protein conformations. These chaperones exhibit a wide range of specificities for certain amino acid sequences and structural features, but in most cases these are hydrophobic sequences that are not exposed in the native protein conformation [11]. Thus, by binding to exposed aberrant sequences, the HSPs play a primary surveillance function in PQC [12].

The chaperones that participate broadly in protein folding and refolding, such as the HSP70s, HSP90s and the chaperonins (HSP60s), are multicomponent molecular machines. They promote *de-novo* folding of nascent proteins as well as refolding of mature misfolded proteins. This is achieved through cycles of ATP binding—ATP hydrolysis and ADP release, mediated by co-chaperones, such as Hsp40s and nucleotide exchange factors (NEFs). Transient chaperone binding blocks aggregation, while ATP-triggered chaperone release allows folding to continue. Repetitive cycles of chaperone binding and dissociation proceed until the native protein conformation is attained [10]. However, when refolding fails, aberrant proteins are degraded by the UPS [13] or, when stable protein aggregates form, by autophagy [2].

In all eukaryotes, the cellular PQC pathway recruits the UPS to dispose of proteins that are beyond repair. UPS-mediated degradation is facilitated by conjugation of Ub molecules (polyUb) to a degradation substrate. Ubiquitin conjugation is mediated by the Ub-ligation system where the Ub conjugating enzymes (E2s) catalyze the addition of Ub molecules to lysine residues of target proteins initially bound to an E3 ligase, the substrate recognition subunit of the Ub-ligation system [14,15]. PolyUb conjugates subsequently provide a signal for substrate degradation by the 26S proteasome.

The large number of E3 ligases encoded in the various eukaryotic genomes (5% of the predicted genes) reflects their ability to discern degradation substrates within the enormous diversity of cellular proteins. Substrate recognition by E3s is highly regulated and selective, thus it occasionally requires auxiliary proteins, among which HSPs are the most prominent. A role for molecular chaperones in UPS-mediated degradation was initially proposed by Ciechanover and colleagues who demonstrated that the *in vitro* ubiquitylation of several proteasome substrates requires the activity of the mammalian heat shock cognate protein 70 (HSC70) [16]. Since then, it became apparent that the majority of E3 ligase complexes of the PQC pathway cooperate with chaperones during proteolysis [17]. However, the nature and extent of the association between E3 ligases and the various HSPs is highly specific and the governing rules for their interaction with PQC-dependent substrates are largely unknown [17].

In addition to their requirement for substrate recognition, HSPs also function downstream to protein-Ub conjugation, as escort factors that deliver and/or dock the Ub-protein conjugates to the proteasome. This chaperone function is particularly important to prevent the formation of ubiquitylated protein aggregates that accumulate in their absence [18,19]. Indeed, ubiquitylated proteins often accumulate within disease related aggregates, emphasizing the additional importance of chaperones downstream to the recognition and ubiquitylation steps [20].

As the roles attributed to chaperones are continually expanding, it becomes apparent that they likely accompany some proteins throughout their life cycle: From folding of a nascent protein to its ultimate demise [21,22,23,24,25]. Yet, our knowledge of the mechanisms that regulate HSPs and in particular, the signals that switch between the different chaperone functions is limited. In this review, we focus on the diverse roles of heat-induced chaperones in targeting misfolded proteins to the proteasome and the consequences of their compromised activity. We provide specific examples to illustrate current models and perceptions and further discuss some of the challenges yet to be solved, in order to better understand the roles and mode of action of chaperones in Ub-mediated proteasomal degradation.

## 2. Chaperone-Assisted Proteasomal Degradation: Hsp40/70s’ Role in Presenting Misfolded Substrates to the UPS

**Figure 2:**
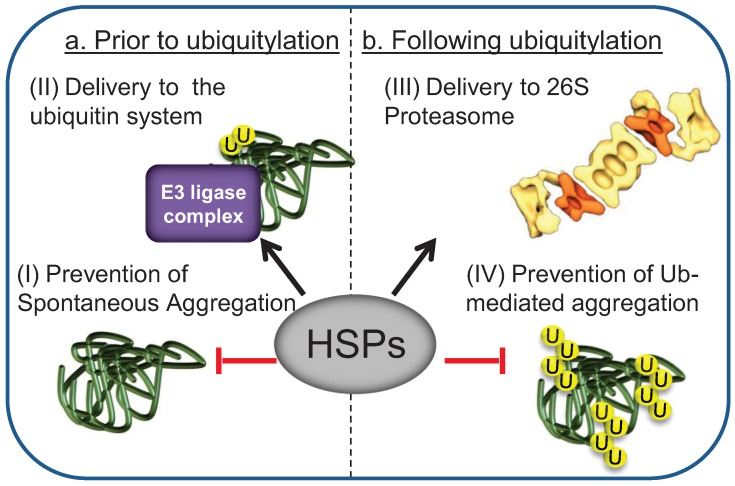
The Hsp70 machinery functions in multiple steps during Ub-mediated proteasomal degradation. (**a**) Prior to ubiquitylation: (I) Heat shock protein (HSP) activity maintains the solubility of misfolded proteins by shielding exposed hydrophobic features; (II) HSP binding targets the substrate to the ubiquitylation complex; (**b**) Following ubiquitylation: (III) HSPs assist delivery of the ubiquitylated substrate to the proteasome; (IV) HSPs may additionally protect ubiquitylated substrates from Ub-mediated sequestration.

The initial step in Ub-mediated degradation of misfolded proteins is their recognition by the E3 ligase complex [26]. The prevailing concept in the field is that targeting of misfolded substrates to the UPS is mediated by the same chaperones that initially recognize protein misfolding and facilitate protein folding and refolding, such as the Hsp70s and their co chaperones, the Hsp40s [27,28]. The Hsp70 chaperone family is comprised of both heat induced Hsp70s and their constitutive counterparts Hsc70s [22]. Since they have overlapping functions, for simplicity, both will be referred to as Hsp70s. Hsp70s, together with their Hsp40 co-chaperones, are the most prominent chaperone families that participate in chaperone-assisted proteasomal degradation of misfolded proteins [29,30]. The diverse roles of these HSPs in the degradation of misfolded proteins by the UPS are summarized in Figure 2.

### 2.1. The Hsp70 Machinery Promotes UPS-Mediated Degradation by Maintaining Misfolded Protein Solubility

To be degraded, a misfolded protein must be maintained in a soluble, non-aggregated state. The yeast Hsp70 chaperone BiP/Kar2 that resides in the lumen of the endoplasmic reticulum (ER), promotes both folding and ER associated proteasomal degradation (ERAD) of secretory proteins, by maintaining them in a soluble state [31,32]. Consequently, when Bip/Kar2 function is inactivated, misfolded secretory proteins such as mutant carboxypeptidase yscY (CPY*) and the non-glycosylated form of pro-α-factor begin to aggregate under conditions that enhance protein misfolding such as elevated temperatures. Under these conditions, the ERAD of these substrates is significantly impaired [33], indicating that the proteasome cannot effectively degrade aggregated proteins [34].

Cytosolic Hsp70s are similarly required for the maintenance of cytosolic, nuclear and membrane-embedded PQC substrates in a soluble state, thus promoting both folding and degradation pathways. Members of the yeast *SSA* family, in particular, were found to promote PQC substrate folding, refolding as well as Ub-mediated degradation. Major *SSA-*dependent degradation pathways include the cytosolic PQC pathway mediated by the E3 ligase Ubr1 as well as the ERAD-cytosolic (ERAD-C) pathway, mediated by the ER/Nuclear envelope embedded E3 Doa10 [35,36,37,38].

**Figure 3:**
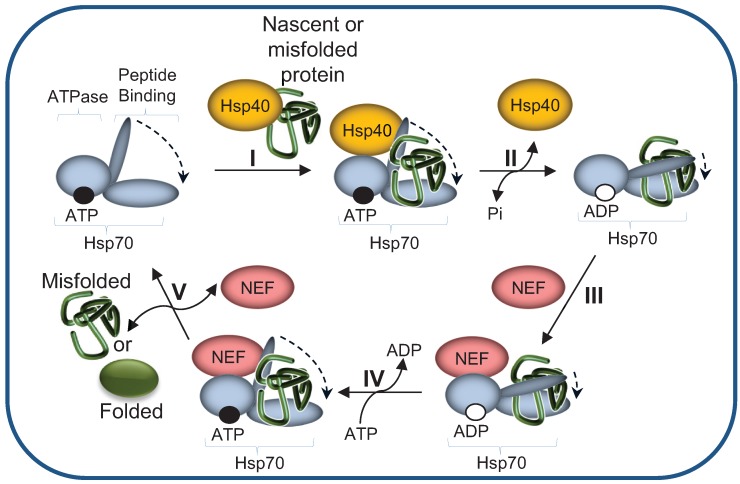
The Hsp70 machinery reaction cycle. (**I**) Hsp40 mediates the delivery of nascent or misfolded proteins to ATP-bound Hsp70; (**II**) Hydrolysis of ATP to ADP, accelerated by the Hsp40, results in Hsp70 conformational change: the α Helical “lid” of the peptide binding domain “closes”, leading to tight binding of the substrate. Hsp40 dissociates from Hsp70; (**III**) NEF (Nucleotide exchange factor) binds to Hsp70, catalyzing the dissociation of ADP (**IV**); (**V**) ATP binds to the Hsp70’s ATPase domain, inducing “opening” of the “lid”, thereby enabling substrate release.

What determines the selection of the degradation pathway in favor of the refolding pathway is not clear. One possibility is that a kinetically controlled triage mechanism determines whether a protein acquires a functional life or is degraded. Another possibility is that substrates exposing common structural degradation motifs are actively targeted to the degradation machinery by specific recognition factors. As Hsp70s reaction cycle requires a network of co-chaperones (Figure 3), dedicated co-chaperones may facilitate the targeting of misfolded proteins to the degradation machinery. We next discuss the emerging possibilities.

### 2.2. Hsp40 Co-Chaperones Are Essential Co-Factors of Hsp70s

The function of Hsp70s requires the cooperation of 40-kDa co-chaperones, also termed J-domain proteins (named after the *E. coli* DnaJ protein). Hsp40s show a substantial degree of sequence and structural heterogeneity, consistent with the requirement to regulate the highly diverse Hsp70 functions [27]. Hsp40s stimulate the ATPase activity of Hsp70s, thereby facilitating substrate capture (Figure 3) [39]. Thus, Hsp40 activity is essential for Hsp70 mediated degradation pathways. For example, in the yeast ER, the luminal Hsp40s Jem1 and Scj1 cooperate with the Hsp70 chaperone Bip/Kar2 to maintain misfolded substrates soluble and to assist in their translocation into the cytosol, where they are subjected to UPS-mediated degradation [33,40]. The cytosolic yeast Hsp40 Ydj1 similarly cooperates with members of the SSA family of Hsp70s to facilitate the folding of nascent polypeptides and prevent protein aggregation [41,42]. Goldberg and colleagues initially demonstrated the essential requirement for Ydj1 in the degradation of short-lived and abnormal proteins [43]. Since then, several studies have established the requirement for Ydj1 in the early steps of proteasomal degradation, mainly as a factor essential for the maintenance of substrate solubility prior to ubiquitylation [35,38,42].

## 3. HSPs Mediate the Interaction of Misfolded Substrates with E3 Ligase Ubiquitylation Complexes

### 3.1. The E3 Ub Ligase CHIP Directly Binds Hsp70-Substrate Complexes

Assigning specific functions to Hsp40/70s in misfolded protein degradation is often difficult, as intracellular manipulation of HSPs activity might result in pleiotropic effects due to their essential and diverse cellular functions. Nevertheless, the discovery of a direct link between Hsp70 chaperones and the E3 ligase termed CHIP (C-terminus of heat-shock cognate 70 stress protein-interacting protein) provided invaluable insights about the role of chaperones in UPS-mediated degradation. Mammalian CHIP is a bifunctional enzyme acting as an E3 Ub ligase as well as Hsp70/90 binding partner [44,45]. Hsp70s bind to the N-terminal tetratricopeptide repeat (TPR) domain of CHIP via a conserved C-terminal EEVD motif [46]. By interacting with both misfolded substrates and CHIP, Hsp70s act as substrate recognition factors. Subsequently, CHIP mediates polyubiquitylation of chaperone substrates by recruiting E2 enzymes that bind to its C-terminal catalytic U-box domain [47]. CHIP substrates include clinically-important proteins, among them the glucocorticoid receptor [44], the receptor tyrosine kinase avian erythroblastosis oncogene B-2 (ErbB2) [48], the Alzheimer’s disease related protein tau [49] and the cystic fibrosis transmembrane conductance regulator (CFTR) [50]. As Hsp70s are integral participants of the CHIP E3 ligase complex, they escort CHIP substrates all the way from the initial recognition to the proteasome.

### 3.2. Dedicated Co-Chaperones Direct a Slow-Folding Protein to the E3 Ligase Complex

The large size and slow folding kinetics of the polytopic membrane protein CFTR provides an excellent model to study the functional conversion of molecular chaperones from protein-folding machines into degradation factors. Loss-of-function mutations in CFTR are responsible for the fatal disease cystic fibrosis, of which the most studied is a deletion of a phenylalanine at position 508 (∆F508) that abolishes CFTR folding and plasma membrane expression [51]. Wild type CFTR attains its native conformation in the ER membrane prior to transfer to the plasma membrane where it functions as a chloride channel [52]. The slow folding process of the large cytosolic domains of both the wild type and mutant CFTR is subjected to CHIP/Hsp70 quality control [53,54]. CHIP-mediated ubiquitylation directs unfolded CFTR to the proteasome [50,54], while inhibition of ubiquitylation allows CFTR folding to proceed. This ubiquitylation-dependent triage decision is regulated by co-chaperones of the NEF family. NEFs stimulate the discharge of ADP from Hsp70, thereby triggering substrate release (Figure 3) [55]. HSPBP1 (Hsp70 binding protein 1) and BAG-2 (BCL2-Associated Athanogene 2) interact with the CFTR-CHIP-Hsp70 complex and inhibit ubiquitylation, thus allowing CFTR folding to proceed [56,57,58]*.* In contrast, binding of several other dedicated co-chaperones shifts unfolded CFTR from the folding pathway to UPS-mediated degradation by enhancing the interaction with CHIP or with other PQC E3 ligases, among them RMA1 [54,59], gp78 [59] and MARCH2 [60].

Studies of CFTR processing in yeast revealed that it requires the cytosolic and the ER membrane tail-anchored Hsp40 co-chaperones Ydj1 and Hlj1, respectively [61]. Subsequent studies in mammalian cells further showed that the conserved human Hlj1-ortholog, DNAJB12, induces nascent CFTR degradation by recruiting the Hsp70-CFTR complex to the ER-associated E3 ligase RMA1 [62]. Thus, the binding of different co-chaperones such as BAG-2 and Hlj1/DNAJB12 influence triage decisions by determining the various Hsp70s functions.

### 3.3. Nucleotide Exchange Factors Regulate Hsp70-Mediated Substrate Association with E3 Ligases

**Figure 4:**
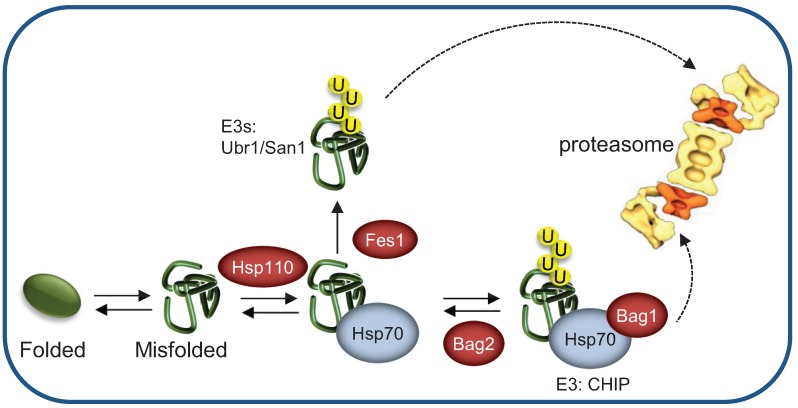
Different nucleotide exchange factors direct the HSP machinery toward refolding or degradation of misfolded substrates. In yeast, most of the cytosolic Hsp70 clients, targeted for degradation are ubiquitylated by the E3 ligases San1/Ubr1. Hsp110 promotes rebinding of misfolded substrates to the Hsp70 machinery, thus assisting protein folding. Conversely, Fes1 stimulates misfolded protein release from the Hsp70 machinery, promoting binding to the E3 ligase. In metazoans, BAG-1 mediates proteasomal degradation by recruiting the Hsp70/CHIP/substrate complex to the proteasome while BAG-2 inhibits CHIP activity, thus promoting refolding.

Binding of NEFs to the CHIP-Hsp70 complex can lead to inhibition of ubiquitylation. The yeast Sse1/Sse2 NEFs of the Hsp110 family are also capable of preventing ubiquitylation by promoting rebinding of misfolded substrates to the Hsp70, thus assisting protein folding (Figure 4) [63,64]. However, other NEFs can enhance ubiquitylation by facilitating the transfer of misfolded proteins from the Hsp70 to specific PQC E3 ligases. The NEF Fes1 was proposed to stimulate misfolded protein binding to the PQC E3 ligase Ubr1, by selectively interacting with misfolded substrates and triggering their release from the Hsp70 (Figure 4) [64]. Consequently, in the absence of Fes1, misfolded proteins fail to undergo polyubiquitylation and aggregate instead. Taken together, these findings indicate that opposing activities of distinct NEFs can determine Hsp70 function toward either the folding or the degradation pathways.

### 3.4. Spatial Regulation of Misfolded Protein Degradation by HSPs

HSPs can also regulate ubiquitylation indirectly, by mediating the subcellular localization of misfolded substrates. The observed cooperation between the cytosolic and nuclear E3 ligases Ubr1 and San1, respectively [65,66], led to the discovery of HSP-dependent compartmentalization as a novel mechanism for PQC regulation. Apparently, Ubr1-mediated ubiquitylation requires HSP chaperones [67] while San1 directly recognizes its misfolded substrates via its intrinsically disordered domains [68]. However, certain cytoplasmic conditions that induce proteotoxic stress lead to degradation of a variety of misfolded cytosolic proteins by San1 in an Hsp70-dependent manner [67]. Hsp70, together with Sse1, mediate the translocation of misfolded substrates from the cytosol to the nucleus where San1 resides and where a significant portion of the cellular proteasome content is found [67,69]. *In-vitro* ubiquitylation studies of the cytosolic PQC substrate NBD2* suggested that Hsp70s can directly assist San1-substrate recognition, possibly by increasing substrate solubility [70]. Thus, Hsp70 are likely to be required in multiple steps during San1-mediated degradation of cytosolic substrates.

Sis1, an essential Hsp40 chaperone in yeast that plays a major function in protecting against prion cytotoxicity [71,72], is also required for nuclear translocation of San1 degradation substrates [73]. Park and colleagues demonstrated that Sis1 mediates the translocation of the partially-misfolded ΔssCPY*-GFP into the nucleus [73]. Upon depletion of Sis1, misfolded proteins cannot enter the nucleus and, as a result, accumulate in cytoplasmic inclusions. Apparently, Sis1, as well as its mammalian ortholog DNAJB1, constantly shuttle between the cytosol and the nucleus and are limiting factors for PQC degradation of partially-misfolded substrates. The fact that the yeast and mammalian Hsp40s function as cytosol-to-nucleus shuttling factors and are both PQC-limiting factors implies that nuclear degradation is a major evolutionary conserved pathway, although the advantage, if any, conferred by such segregation is yet unclear.

### 3.5. Terminally- and Partially-Misfolded Proteins are Targeted for Proteasomal Degradation by Distinct HSP-Dependent Pathways

A common approach in studies of the PQC system has been to employ heterologous overexpression of terminally misfolded proteins, such as those with poly glutamine (polyQ)-extended regions. This approach has several advantages, allowing the direct study of disease related proteins, such as the polyQ huntingtin, associated with Huntington’s disease. However, the use of these substrates does not necessarily mimic the slow, time-dependent accumulation of misfolded proteins, as observed in most neurodegenerative disorders. Therefore, several studies have adopted a different experimental approach, in order to possibly allow for a more accurate recapitulation of the chronic buildup of protein homeostasis stress. These systems follow endogenous UPS substrates, comprised of non-toxic, partially-misfolded proteins that permit degradation/sequestration studies, in the absence of extraneous stress. Newly developed partially misfolded model substrates in yeast, such as GFP*-DegAB* [18,74] or ΔssCPY*-GFP [19,73,75], do not aggregate spontaneously and hence can be used to distinguish different chaperone requirements for the recognition, proteasomal degradation and aggregation of misfolded proteins.

**Figure 5:**
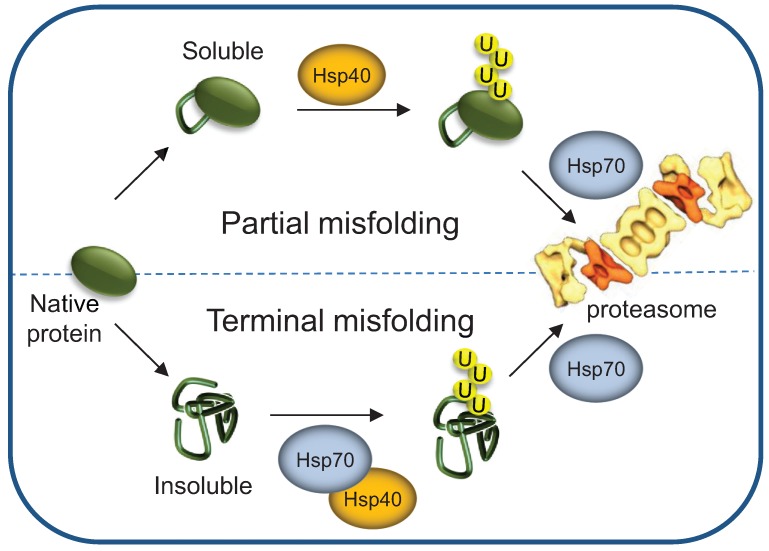
Hsp40/70 requirement is determined by the severity of protein misfolding. Terminally as well as partially misfolded proteins require Hsp40 activity for initial sorting of the ubiquitylation complex. However, only terminally misfolded proteins require the Hsp70s prior to ubiquitylation. The HSP70s may be needed for maintaining misfolded substrate solubility and/or for assisting interaction with the ubiquitylation complex. Still, both terminally and partially misfolded ubiquitylated substrates require Hsp70s activity for 26S proteasome degradation.

Results obtained through studying the fate of endogenous UPS substrates, revealed distinct Hsp70 requirements for Ub conjugation: While Hsp70 is obligatory for terminally misfolded proteins [35,38,42,67,70,76,77] it is redundant for partially misfolded proteins [18,19,74]. These special HSP requirements reflect protein solubility: Substrates such as GFP-*DegAB* or ΔssCPY*-GFP with mild structural perturbation are defective mainly in their tertiary but not secondary structure [18,19,73,74] and remain soluble. In contrast, terminally misfolded proteins, such as prion like polyQ-extended proteins, with extensive loss of secondary structure, are mostly insoluble. Hence, binding of Hsp70 to terminally misfolded proteins protects them from spontaneous aggregation and keeps them accessible to the ubiquitylation machinery (Figure 5).

### 3.6. Hsp40s May Act as Hsp70-Independent Ubiquitylation Factors—the Case of Sis1

While terminally misfolded substrates, such as the yeast Ste6*, require the activity of both Hsp40 and Hsp70 for ubiquitylation [35], E3 recognition of the partially misfolded substrate GFP-*DegAB*, requires only the Hsp40 [18]. We found that the specific interaction between a substrate containing the *DegAB* degron and its cognate E3, Doa10, requires Sis1 activity but is independent of Hsp70s [18]. On the other hand, Sis1 is dispensable for the ubiquitylation of ΔssCPY*-GFP [73]. This was unexpected, since Sis1 is also essential for the translocation of ΔssCPY*-GFP into the nucleus for interaction with San1 [73]. Likely, in the absence of Sis1, ΔssCPY*-GFP can still undergo ubiquitylation via the cytosolic E3 ligase Ubr1 [65,73]. Most importantly, the lack of Hsp70 requirement for ubiquitylation enables the distinction between the roles of Hsp70 upstream and downstream to ubiquitylation. Conceivably, Hsp70s are required for shuttling ubiquitylated substrates to the 26S proteasome (see Section 4).

## 4. HSPs’ Roles as Proteasome Shuttling and Docking Factors

Following ubiquitylation, misfolded substrates still require chaperones to maintain them in a soluble state *en-route* to the proteasome [18,19,78]. For example, an Ub ligase-associated chaperone holdase has been shown to maintain ubiquitylated polypeptides bearing exposed transmembrane domains in a soluble state available for proteasome degradation [78]. Besides shuttling misfolded ubiquitylated substrates to the proteasome, Hsp70s may additionally protect poly-Ub chains from premature trimming by deubiquitylating enzymes and they serve as mediators of 26S proteasome binding.

### 4.1. The Co-Chaperone BAG1 Mediates Binding of Hsp70-Substrate Complexes to the Proteasome

As discussed above (Section 3.1), Hsp70s are required for the recognition of misfolded substrates by the CHIP ubiquitylation complex. Yet, an additional co-chaperone is required for the association of the ubiquitylated protein with the 26S proteasome. Bcl2-associated athanogene 1 (BAG-1) is a human Hsp70-associated NEF that was initially discovered as a binding partner of the apoptosis inhibitor Bcl-2 [79]. Two functional domains of BAG-1 act in concert to ensure the proteasomal degradation of CHIP-ubiquitylated substrates: A Ub-like domain (UBL) of BAG-1 that mediates the association with the 26S proteasome [80,81,82] and a conserved BAG domain that mediates Hsp70 binding and also triggers the timely substrate release from the Hsp70 complex to the proteasome [83]. Consequently, BAG-1 competes with its family member BAG-2 (Section 3.2) in binding to the Hsp70 ATPase domain (Figure 4). Thus, BAG-1 and BAG-2 might be viewed as antagonistic regulators of the CHIP Ub ligase [58]. The BAG-1 function is conserved in evolution. Yeast do not express a CHIP homolog, but fission yeast encompass a BAG-1 ortholog whose BAG and UBL domains are essential for the PQC-degradation of the San1 substrate, mutant kinetochore protein Spc7 [84].

### 4.2. HSJ1 Is a Tissue-Specific Hsp40 Required for Shuttling Substrates to the Proteasome

An additional Hsp40 co-chaperone HSJ1 (DNAJB2) that acts mainly in neurons also facilitates CHIP-substrate interaction with the proteasome [85]. The J-domain of HSJ1 stimulates substrate loading onto the Hsp70 chaperone, while its two Ub-interaction motifs (UIMs) bind polyUb chains on chaperone substrates. HSJ1 binding thus fulfills a dual function that promotes proteasomal targeting and efficient degradation: It prevents ubiquitylated protein aggregation and shields against polyUb chain trimming by Ub hydrolases [85]. HSJ1 was proposed to directly participate in the degradation of CFTR as well as neurodegenerative disease related proteins, such as the amyotrophic lateral sclerosis (ALS) linked mutant protein superoxide dismutase 1 (SOD1) [86] and the expanded polyQ forms of spinocerebellar ataxia type 3-linked protein [87]. As HSJ1 is expressed mainly in neurons, it may comprise a novel escort pathway to the proteasome, specific to neuronal cells. As such, it may constitute a central target for therapeutic approaches in neurodegeneration.

### 4.3. Hsp70 Ubiquitylation Mediates Hsp70-Substrate Complex Docking at the Proteasome

The Hsp70s were identified early on as targets for CHIP-dependent ubiquitylation, composed of short Ub chains that do not affect the chaperones’ stability. Ubiquitylation of Hsp70s was found to be required mainly to facilitate client substrate delivery to the proteasome [47,85,88]. In agreement with that, several ubiquitylation sites in Hsp70s have been mapped. In particular, Lys521 was shown to be ubiquitylated in both yeast and human cells [89,90], suggesting a conserved function in triggering misfolded substrate shuttling to the proteasome. Thus, ubiquitylation of Hsp70s enables their direct binding to diverse Ub-binding domains (UBDs) associated with receptors that are present on the 19S regulatory particle of the proteasome and facilitate substrate–proteasome interaction.

## 5. Chaperones Prevent the Aggregation of Misfolded Degradation Substrates

### 5.1. Hsp70s Cellular Levels Determine the Fate of Polyubiquitylated Proteins

**Figure 6:**
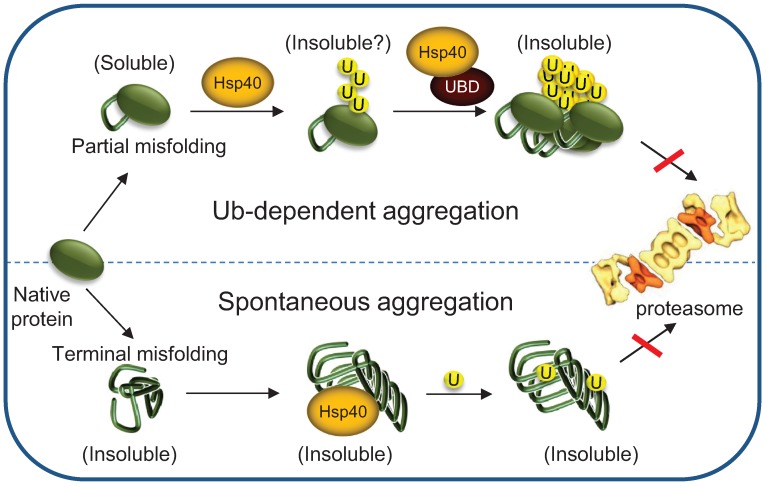
The cellular consequences of misfolded protein aggregation upon Hsp70 depletion. Hsp70s depletion, as happens during ageing, induces spontaneous aggregation of terminally misfolded proteins, enhancing sequestration of molecular chaperones and additional critical factors, and thereby their cytosolic depletion. On the other hand, partially misfolded proteins remain soluble, until they are tagged for degradation by Ub conjugation. However, when Hsp70 levels are limiting, the ubiquitylated substrates are not degraded by the 26S proteasome. Instead, they undergo Ub-mediated sequestration, which may require the association with proteins containing Ub binding domain (UBDs).

As discussed above (Section 2.1), Hsp70 function is critical in order to maintain terminally misfolded proteins in a soluble state for Ub conjugation and proteasomal degradation. Hence, Hsp70 depletion can lead to spontaneous aggregation of terminally-misfolded proteins such as polyglutamine-expended huntingtin protein [91], alpha-synuclein [92] and amyloid beta (1–42) [93], which have been linked to Huntington’s, Parkinson’s and Alzheimer’s diseases, respectively. Surprisingly, Hsp70 depletion also has a remarkable effect on the aggregation of partially misfolded proteins that retain their secondary structure and are intrinsically soluble [18,19,74]. We have recently reported that in Hsp70-depleted cells, Ub-conjugates of a partially misfolded protein are incorporated into aggregates rather than degraded [18], indicating that Hsp70 activity is required to protect ubiquitylated proteins from sequestration. The dependence on ubiquitylation suggests that aggregation of ubiquitylated proteins is an active process, possibly involving polyUb recognition factors (Figure 6). This emerging pathway requires future studies in order to corroborate the role of polyUb conjugates in recruiting sequestration factors, as well as Hsp70 function in protecting ubiquitylated proteins from these factors. Interestingly, a gradual depletion of Hsp70 expression is observed during aging in *Caenorhabditis elegans* and *Drosophila melanogaster.* This depletion is associated with diminished protein homeostasis capacity [94,95]. As the most common risk factor for neurodegeneration is aging [96,97], the emerging role of Hsp70 machinery in protecting ubiquitylated proteins from sequestration may explain the increased appearance of inclusion bodies late in life, and why Ub is so abundant in these disease related inclusions.

### 5.2. Implications to Protein Misfolding and Degenerative Diseases

Analyses of chaperone-assisted degradation have highlighted the central role of the HSPs in maintaining the delicate balance between the protection of folding intermediates from aggregation and the efficient clearance of misfolded species that pose a threat to cell viability.

Impaired protein homeostasis is prevalent in neurodegenerative diseases, malignancies and cardiomyopathies as well as during aging [20,95,98,99,100,101,102]. Common to these conditions is a substantial buildup of damaged proteins and intracellular protein aggregates. Alleviating the damaged protein burden in these conditions by modulating PQC may thus hold a therapeutic promise. For example, elevated levels of the Rpn6 regulatory subunit of the proteasome were found to increase the lifespan of worms and human embryonic stem cells [103,104]. However, the expression of Rpn6 in human embryonic stem cells actually decreased upon differentiation [104]. Thus, it is unclear whether Rpn6 upregulation will be feasible and effective in post-mitotic mammalian systems. Furthermore, upregulation of the proteasome was observed in a wide variety of malignancies and was shown to increase breast cancer metastasis [105]. Manipulation of Hsp70 activity similarly led to contradicting results: In addition to positive clinical effects of Hsp70 overexpression, such as increased resistance to cardiomyopathies [106] and a delay in progression of neurodegeneration in a mouse model of Spinocerebellar ataxia type 1 (SCA1) [107], increased Hsp70 levels could have negative clinical effects, such as promoting cancer cells survival [108,109]. One implication of the studies reviewed here is that such manipulations need to be more selective and should target the components providing specificity to the degradation machinery, namely the PQC E3 Ub ligases and the Hsp70 co-chaperones: the Hsp40s as well as the NEFs.

A proof of concept for the validity of targeting Hsp40s for neurodegeneration therapy is provided by studies of the human Hsp40 chaperone HSJ1 whose overexpression in ALS mice model ameliorated the disease state. HSJ1 specifically elevates mutant SOD1 ubiquitylation and reduces its aggregation, hence improving motor neuron survival [86]. The overexpression of HSJ1 also reduces mutant Huntingtin aggregation in mouse brain, in a manner that is dependent on both its J and UIM domains [110]. Importantly, HSJ1 selectively binds to the mutant but not to the wild type proteins, emphasizing its selective function.

A hypothetical approach to correcting the cystic fibrosis defect been proposed, based on downregulation of the E3 ligases RMA1 and CHIP, which enables mutant CFTR to complete its folding without being degraded. However, the combined inhibition of both RMA1 and CHIP activities was required in order to compensate for the various folding defects caused by the F508 deletion, as CHIP and RMA1 recognize different regions of CFTR [111]. This underscores the specificity of the E3 Ub ligases and exemplifies the potential complexities involved in this type of approach.

The identification of specific E3s, Hsp40s and novel sequestration factors that interact with disease-associated misfolded proteins and elucidating their function therein, continue to be a major objective of current and future studies. The next challenge will be to develop efficient methods to regulate the levels and/or activities of specific PQC components, in order to maintain balanced protein homeostasis.

## 6. Conclusions and Perspectives

In this review, we have focused on the multiple roles of Hsp70 chaperones and their co-chaperones, the Hsp40s, as regulators of misfolded protein elimination by the UPS. Although in many instances the functions of the Hsp40/70s are inseparable, we have endeavored to highlight the points in which their functions diverge, in order to understand their underlying mechanisms of action. The data reviewed herein indicates that Hsp70s are required at multiple steps during misfolded protein degradation, from protection against aggregation to the delivery of ubiquitylated substrates to the proteasome for degradation. The emerging data on Hsp40 functions suggest that they operate as part of multi-protein complexes, engaged in the recognition of misfolded proteins and their delivery to the E3-ligase and ultimately to the proteasome. Hsp40s can additionally target misfolded substrates to designated inclusion bodies, when Hsp70 activity declines.

A major question that remains is how HSPs switch from protein-folding machines into degradation or sequestration-promoting factors and what are the underlying regulatory mechanisms. One way to approach this question is by focusing further research on model degradation substrates with minor structural perturbations that do not affect protein solubility, rather than on severely misfolded proteins with an innate tendency to undergo aggregation. Partially-misfolded proteins are more likely to represent the majority of PQC degradation substrates encountered by the proteasome in living cells. These substrates can be particularly useful for identifying factors affecting the triage decision under conditions where the UPS system is overwhelmed or begins to deteriorate, as occurs in ageing.
